# Current practices in spatial analysis of cancer data: data characteristics and data sources for geographic studies of cancer

**DOI:** 10.1186/1476-072X-3-28

**Published:** 2004-12-01

**Authors:** Francis P Boscoe, Mary H Ward, Peggy Reynolds

**Affiliations:** 1 New York State Cancer Registry, New York State Department of Health, Albany, NY, USA; 2 Occupational and Environmental Epidemiology Branch, Division of Cancer Epidemiology and Genetics, National Cancer Institute, NIH, DHHS, Bethesda, MD, USA; 3 California Department of Health Services, Environmental Health Investigations Branch, Oakland, CA, USA

## Abstract

The use of spatially referenced data in cancer studies is gaining in prominence, fueled by the development and availability of spatial analytic tools and the broadening recognition of the linkages between geography and health. We provide an overview of some of the unique characteristics of spatial data, followed by an account of the major types and sources of data used in the spatial analysis of cancer, including data from cancer registries, population data, health surveys, environmental data, and remote sensing data. We cite numerous examples of recent studies that have used these data, with a focus on etiological research.

## Introduction

Understanding the spatial patterns of diseases in a population can provide insight as to their causes and controls. Indeed, this notion is at the very root of the field of epidemiology [1]. The recent explosion in data gathering, linkage and analysis capabilities fostered by computing technology, particularly geographic information systems (GIS), has greatly improved the ability to measure and assess these patterns. Large and complex georeferenced data sets are now readily available through Spatial Data Clearinghouses, facilitating analyses by researchers unaffiliated with the government agencies that have historically controlled data access. Meanwhile, increasingly sophisticated statistical tools have evolved to keep pace with the increased data availability and computing power.

The purpose of this article is to provide an overview of spatial data and its relevance to population-based cancer surveillance and research in the United States as of 2004. We begin by discussing a number of the distinctive characteristics of spatial data, which can sometimes hinder efforts to understand cancer etiology. We then proceed to describe the kinds of data sets that are available, accompanied by a survey of some applications using these data. Finally, we discuss several ongoing efforts to provide central repositories of geospatial data. Given the vast scope of cancer research taking place worldwide, our survey is necessarily partial, and we have chosen to emphasize etiology over other research themes with spatial dimensions, such as patterns of treatment or access to care [2].

## Qualities of spatial data

Spatial data refer to data with locational attributes. Most commonly, locations are given in Cartesian coordinates referenced to the earth's surface. These coordinates may describe points, lines, areas or volumes. This need not be the only spatial framework; "relative spaces" may be defined in which distance is defined in terms of some other attribute, such as sociodemographic similarly or connectedness along transportation networks [3,4]. Spatial data have special qualities that require specialized statistical techniques and modeling approaches. A complete discussion of these special qualities is well beyond the scope of this article, but here we describe a number of the more compelling and recurring themes. For a focused discussion on the limitations on analysis that these data characteristics impose, see the companion piece to this article, "Current Practices in Spatial Analysis of Cancer Data: Flies in the Ointment, Or, The Limitations of Spatial Analysis" [5].

Individual humans represent the basic unit of spatial analysis in cancer research. Individuals are categorized as either having or not having a disease or attribute of a disease, and are assigned coordinates corresponding to the location of their place of residence, a technique known as geocoding. As with all measurements, geocoding involves some error. A growing body of literature is exploring the nature of this error and its potential to bias epidemiologic studies [6]. Among the topics that have been investigated are systematic problems with geographic reference files [7], the ramifications of different geocoding algorithms [8], positional accuracy [9] and how to handle non-residential addresses, such as rented post office boxes [10].

Assigning individuals to their place of residence also poses problems, although this is usually the only locational information that is available. Often the goal of a geographic analysis is to identify a common environmental exposure in a population, but exposures that are occupational or recreational may not necessarily reveal themselves in a residential analysis. Also, given the long latency period for many cancers and the mobility of the American population, the relevant exposure may be associated with a prior address. Difficult-to-measure behavioral risk factors such as smoking and diet often further confound attempts at geographic analysis.

Owing to confidentiality restrictions, researchers outside of central cancer registries typically do not have access to address-level data. In such instances, case data are aggregated by some functional or political unit such as census tract, county or ZIP code. Even when the case data are geocoded, population data must be aggregated, at least to the level of the census block, which is the smallest unit for which any population information is available. Knowing that there are four women with breast cancer living on the same street is not sufficient, by itself, to draw conclusions about whether the street displays an unusual incidence pattern; one must also know the number and ages of women without breast cancer on the same street. Short of conducting one's own thorough door-to-door census, this question cannot be answered, except by aggregating the street segments into blocks. When additional variables, such as measures of income or education, or also of interest, then still larger analytical units must be chosen.

The necessity for aggregating spatial data raises a whole set of analytic issues regarding the extent to which the act of aggregating introduces error and bias. It is theoretically possible to achieve dramatically different, even contradictory results, simply as a consequence of aggregating the data in a different fashion [11]. This is true not only for aggregations at different spatial scales, but also different aggregations at the same scale. Geographers have termed this the "modifiable areal unit problem" [12]. A special case of the modifiable areal unit problem is the ecological inference problem, which specifically refers to the lack of congruity between associations found in aggregated and individual-level data.

In practice, well-chosen scales and groupings can minimize the modifiable areal unit problem and allow reasonable consistency between aggregated and individual-level results [13]. There will always be exceptions to this, however, as evidenced by the many studies attempting to relate low-level indoor radon concentrations with lung cancer incidence. Individual-level studies have repeatedly found a positive correlation, while area-level studies have found a negative correlation at low radon levels [14-16]. Despite a general appreciation of how these discrepant results represent an example of aggregation bias, there is still active debate over what these results say about low-level radon risk [17,18].

Often analyses need to be performed on data that was collected at different spatial scales, such as a study using cancer cases aggregated by ZIP code and modeled air pollutant data at the census tract level. The resulting scale-translation problem is a recurring one that has inspired many independent solutions, and is known variously as areal interpolation, the polygon overlay problem, and the problem of inference with spatially misaligned data, among other terms [19]. The most naïve solution to this problem is to assume that each measured value is homogeneous within each spatial unit. Under this assumption, using our example, a ZIP code that is coincident with four census tracts would be broken into four polygons, each having the same cancer rate but different air pollutant values. More sophisticated cartographic overlay techniques have been developed that involve using covariate information to infer variation within spatial units. To date, these techniques have been primarily applied toward estimating population surfaces rather than cancer or other disease rate surfaces [20,21]. Hierarchical Bayesian and multi-level logit models have also shown promise [22-25].

Spatial autocorrelation is another distinctive quality of spatial data that requires the use of specialized analytic methods. Spatial autocorrelation is the tendency for nearby observations to have correlated attribute values. For most data sets involving the distribution of human populations and their characteristics, spatial autocorrelation is positive, meaning that neighboring individuals tend to have similar characteristics. Understanding the characteristics and qualities of spatial autocorrelation is essential to adequately model and interpret geographic patterns. For example, it is not appropriate to perform ordinary least squares regression on spatial data, because the presence of spatial autocorrelation means that the observations are not independent. Performing such a regression generally results in downwardly biased estimations of variance, which yields overstated levels of significance. In general, spatially autocorrelated data is less informative in a model than uncorrelated data. There is an ample literature on assessing and properly accounting for spatial autocorrelation in geographic analysis [26,27].

A final critically important characteristic of spatial data is spatial nonstationarity, or the tendency for relationships between and among variables to vary by geographic location [28]. First-order or strong stationarity refers to the degree to which measured values vary spatially, while second-order or weak stationarity refers to the degree to which the uncertainties in these measured values vary spatially. So-called global statistics ignore nonstationarity, suggesting that relationships across space are constant. The simple linear equation that has traditionally been used to express the relationship between rainfall and altitude is a well-known example. Local statistics, in contrast, take nonstationarity into account, at least first-order nonstationarity. Brunsdon et al. [29] used the technique of geographically weighted regression to demonstrate that both the slope and intercept of the rainfall-altitude equation vary considerably in space. The range and breadth of local statistics has seen rapid growth in recent years [27,30].

Local statistics are less adept at accounting for second-order nonstationarity. Indeed, many of these methods require the assumption of constant variance across space. Because of the uneven distribution of human populations, this assumption is seldom met for health data. Specifically, disease rates in areas with smaller numbers of cases are more variable than those in areas with larger numbers of cases, a property that has also been termed "variance instability" [31]. Variance instability is particularly pervasive on maps, since it is extremely difficult to design a map that is not visually biased toward either sparsely populated or densely populated areas [32,33]. A simple example is the tendency for rural counties to contain disproportionate numbers of unusually high or unusually low disease rates and thus visually dominate a choropleth map. The problem is compounded by the tendency of such counties to be large in size; for these reasons, maps of United States counties are often visually dominated by such states as Idaho, Nevada and Wyoming.

Efforts to include information about data uncertainty have shown promise, but have not seen widespread use [34]. One common way of addressing this problem is to produce smoothed maps, whereby the rate for a given area is influenced by the rates of neighboring areas. There are many algorithms available to accomplish this [35], ranging from conceptually straightforward spatial filters [36] to computationally-intensive Bayesian approaches [37,38]. Properly accounting for second-order spatial nonstationarity in maps and models remains an active research area.

## Types and sources of data

In this section we described the primary types and sources of data most frequently used in the geographic analysis of cancer, along with examples of their application. These are summarized in Table 1.

**Table 1:** Sources of Cancer Registry Data

Dataset name	Source Agency	URL	Geographic Resolution
SEER*Stat, Cancer Mortality Maps and Graphs, State Cancer Profiles	National Cancer Institute		County
Florida Cancer Data System	University of Miami School of Medicine		County
Cancer Incidence and Mortality Rates in Kentucky	Kentucky Cancer Registry		County
New York Cancer Incidence by ZIP code	NYS Department of Health		ZIP code

### 1. Cancer registries

A cancer registry is a data collection system that tracks cancer cases that have been diagnosed or treated in a specific institution or geographic area. Cancer registries typically collect information from medical records provided by hospitals, doctors, other care facilities, medical laboratories, and/or insurers. Data collected by cancer registries is stored under secure conditions so as to protect confidentiality.

Historically, observed geographic differences in cancer incidence have been of great interest in trying to understand more about factors which may influence risk of these diseases. Such differences have served as the basis for studies of migrant populations and acculturation differences in migrant groups. They have been possible because cancer is one of the few chronic diseases for which high quality population-based disease surveillance systems have been in place for many years in many countries of the world.

Cancer registry data has been widely applied toward the production of cancer atlases [39], studies analyzing the spatial distribution of particular cancer sites [40], and studies assessing spatial clustering [41]. Most recently, cancer studies have been undertaken which build on the combined resources of cancer registry data and increasingly available GIS tools. Because address at diagnosis is available for most registry cases it can be geocoded and integrated in a GIS with social and environmental attribute information available at a variety of geographic scales. Examples of such approaches include studies of childhood cancer which examine rate differences in areas of low versus intense agricultural pesticide use [42], heavy traffic patterns [43], or high air pollution [44]. Alternatively, cancer registry data can serve to identify population-based cases for studies using case-control or cohort designs, which can in turn be integrated into a GIS for area attribute data. Examples of this approach include case-control studies of childhood leukemia and traffic patterns [45-48]. and a studies of breast cancer incidence associated with residence in high pesticide use areas in a large case-control study [49,50]. and in a large cohort study [51].

For these types of studies, cancer registry data offer both a number of strengths and limitations. Primary strengths include the comprehensiveness of geographic coverage, detailed information on disease subgroups, and rich covariable information on demographic characteristics for each newly diagnosed case of cancer. Because registry data are abstracted from medical records and reflect information for a snapshot in time, primary limitations include the lack of historical information on various factors of potential interest including residential mobility and relevant personal behaviors. Cancer registries typically collect information on the residential address for individuals newly diagnosed with cancer at the time of that diagnosis. Since this is the locational information which serves as the basis for national and international statistics on area cancer rates, it is also useful for looking at area characteristics associated with rate differences, although inferences about etiologic associations are limited for these long latency diseases, and even more so for residentially mobile populations.

The Surveillance, Epidemiology and End Results (SEER) program of the National Cancer Institute (NCI) offers county-level incidence data for its member registries, which cover part or all of eight states, through its SEER*Stat software. Because it provides direct access to individual cancer records, users must first sign a data access agreement. County-level mortality data for the entire United States, collected and maintained by the National Center for Health Statistics (NCHS), is also accessible through SEER*Stat. These data include all causes of death, not just cancer deaths. Selected county-level cancer data may also be accessed through the NCI's Cancer Mortality Maps and Graphs and State Cancer Profiles web sites. The latter was launched in 2003 and contains a host of innovative statistical graphics. Many individual state registries also offer additional geographically referenced data. For example, the Florida Cancer Data System web site allows users to generate a variety of county- and facility-level tables and county-level maps on demand. The Kentucky Cancer Registry also offers a county-level mapping application. New York State offers a limited set of ZIP code level data for the four most common cancer types in the mid-1990s. Currently, county-level cancer incidence data is not available nationally.

### 2. Population data

The United States Census Bureau is the principal source of data on the entire population; most countries have comparable agencies. Since cancer rates are calculated by dividing the number of cases by the number of people at risk, census data is frequently referred to as "denominator data". Census data are readily available in electronic format through the Census Bureau web site, . Data are available in three basic formats. American FactFinder is a web-based application that allows users to drill down through geographic levels to find data tables of interest. It is most useful for data queries that are well-focused. Data may also be downloaded through an ftp server. This method obtains raw text files that require computer code to be written before the data can be easily accessed or manipulated. This method is most useful for users with large data needs who are in possession of some database programming skills. The third approach is to purchase DVDs from the Census Bureau's Customer Service center. The DVDs allow data output in many spreadsheet and database formats, facilitating the ability for users to process and analyze the data. There are also a large number of third-party vendors who offer similar services [52].

The four primary data files emanating from the 2000 census are named Summary File 1 through Summary File 4 (SF1–SF4). SF1 contains population counts by age, sex, race and ethnicity and basic housing characteristic information for the entire population, to the block level. SF2 contains similar information, detailed for ethnic subgroups, American Indian and Alaska Native tribes, and multiple-race individuals. These data are suppressed when the total number of individuals in a given geographic unit totals fewer than 100. SF3 contains detailed housing, demographic, and socioeconomic data to the census block group or census tract level, based on a long form that was sent to one in six households. Census block groups have an optimal population size of 1,500 and census tracts have an optimal population size of 4,000, though in practice populations vary widely. SF4 contains the same information as SF3 for detailed race and ethnic groups, with the same suppression rule as SF2. In addition to these four primary data files, the Census Bureau also provides digital cartographic boundary files for political entities in the country, as well as approximations of postal code boundaries known as ZIP code tabulation areas (ZCTAs).

The Census Bureau also conducts the American Community Survey (ACS), an ongoing survey designed to reach 3 million households each year nationwide. The goal of this survey is to allow the publication of detailed demographic and socioeconomic information more often than once a decade. Data for geographic units totaling more than 65,000 people will be released annually, while data for smaller geographic units will be based on either a three or five year moving average. It will replace the census long form, which will not be administered in 2010. There will undoubtedly be a challenging adjustment period as public health researchers begin to use ACS data.

At present, the level of information available for intercensal time points is quite limited, and derives from Census Bureau estimates at the state or county level. These estimates are used in the calculation of cancer rates by federal and state agencies, although some research has shown that they are not especially reliable, particularly county-level estimates for specific race groups [53]. Various private vendors publish intercensal estimates for areas smaller than counties, though it is impossible to verify their accuracy. Since many vendors use the Census Bureau estimates as controls (for example, vendor estimates of ZIP code populations in a county must add to the Census Bureau estimate for that county), vendor estimates necessarily suffer from the same limitations as the Census Bureau estimates. Finally, some state governments publish their own population estimates. Generally, these estimates are thought to represent improvements over the Census Bureau estimates because of higher levels of local knowledge and a broader use of data sources. We are unaware of any independent efforts to evaluate these claims, however. Examples include the population estimates and projections published by the California Department of Finance, and those by the Epidemiology Program of the Cancer Research Center of Hawaii. The latter population estimates were developed in response to a concern that the Native Hawaiian population was substantially undercounted in previous censuses, and are used by the NCI in calculating national cancer rates.

The 2000 census allowed respondents to select more than one race, although cancer data are only beginning to be collected in this manner. As a result, population data from 2000 must be "bridged" back to the earlier single-race categories to allow comparisons with earlier data. NCHS developed a sophisticated bridging algorithm taking into account age, sex, distribution of single-race groups within counties, and other covariates [54]. This algorithm is reflected in the 1991–2003 population projections and estimates that are published on the NCI web site and included in their statistical software. The Census Bureau itself uses a simpler algorithm in its estimates, allocating equal proportions of each multiple-race combination to the constituent single races [55]. Given the multiplicity of population estimates and methods for calculating them that are available, it is important to be aware of the sources of these data, and how they may influence the confidence associated with a particular research result. This is especially true for small-area analyses, where uncertainties are highest.

In addition to the issues noted above, it is important to realize that even the decennial census counts are not as accurate as popularly believed. The census represents an attempt to enumerate the population as of a single date, but invariably some people are missed or double-counted. These undercounts and overcounts are differential by race, socioeconomic status, and geographic area, potentially biasing cancer rates [56,57].

Countless epidemiologic and geographic studies make use of census data in some capacity, including most studies that report cancer rates for geographic areas. It is also quite common to use census data where individual-level data are not available, particularly for indicators of socioeconomic status [58-60], educational attainment [61] and housing characteristics [7]. Table 2 summarizes the population data sources described in this section.

**Table 2:** Sources of Population Data

Dataset name	Source Agency	URL	Geographic Resolution
2000 Census Summary Files 1–4	US Census Bureau		Census Tract, Block Group or Block (varies by data element)
American Community Survey	US Census Bureau		Areas with populations >65,000
E-1 City/County Population Estimates, with Annual Percent Change	California Department of Finance		City/County
US Population Data, 1969–2001	National Cancer Institute		County

### 3. Surveys

In addition to the Census Bureau as a primary source of sociodemographic attribute data, special survey data can provide valuable information on these characteristics for population groups in some areas. Perhaps one of the best known such surveys is the CDC-sponsored Behavioral Risk Factor Surveillance System (BRFSS), which is touted as the "world's largest telephone survey". Designed in the 1980s to track trends in behavioral risk factors at the state level, this ongoing system of national surveys also provides subarea and subgroup information within some of the larger states. Some researchers have estimated county-level behavioral risk factor prevalence by combining the statewide BRFSS data with county-level demographic data [62,63]. A mapping application to view BRFSS response data at the state and metropolitan level is also available .

Another well-known national survey is the NCHS's National Health and Nutrition Examination Survey (NHANES), which has been in place since 1960 and combines questionnaire information with a national physical examination and biomonitoring program. NCHS also sponsors a National Health Care Survey (NHCS), a National Health Interview Survey (NHIS), a National Immunization Survey (NIS), and a National Survey of Family Growth (NSFG). Similarly designed large-scale efforts to track temporal and area differences for targeted health behaviors within a state include California's Tobacco Survey, Women's Health Survey, and Health Information Survey (Table 3).

**Table 3:** Sources of survey data. Survey data recorded at the ZIP code level are designed to give valid estimates of risk factor distributions at the State level.

Dataset name	Source Agency	URL	Geographic Resolution
Behavioral Risk Factors Surveillance Survey (BRFSS)	Centers for Disease Control		ZIP code
National Health and Nutrition Examination Survey (NHANES), National Health Care Survey (NHCS), National Health Interview Survey (NHIS), National Immunization Survey (NIS), National Survey of Family Growth (NSFG).	National Center for Health Statistics		Metropolitan Statistical Area, National Region
California Tobacco Survey	California Department of Health Services		ZIP code
California Women's Health Survey	California Department of Health Services		ZIP code
California Health Information Survey	UCLA Center for Health Policy Research		ZIP code

Although population survey data has not been extensively incorporated into GIS studies to date, these resources may in the future provide some opportunity to characterize regional differences in behavioral risk profiles targeted for specific health outcomes.

### 4. Environmental data

Over the past several decades there has been a large increase in the availability of spatially registered environmental data in the United States and other countries. Much of these data have been collected as a result of environmental regulations or government-funded research efforts. Examples of US programs to collect spatial data on concentrations or releases of pollutants in the environment include the United States Geological Survey (USGS) National Assessment of Water Quality program (NAWQA) , the Environmental Protection Agency (EPA) National Air Toxics Assessment database , and EPA's Toxic Release Inventory program . EPA has organized environmental data in an umbrella database called Envirofacts Data Warehouse . Some states have extensive efforts to collect additional environmental data. An example is California's Pesticide Use Reporting program ) that requires reporting of all agricultural pesticide use at the level of Public Land Survey System sections (a unit approximately one square mile in area).

There are several issues to consider in using these data for assigning "exposure" in epidemiologic studies. Monitoring data collected for regulatory purposes should be carefully evaluated for its usefulness for estimating individual exposures. The fate and transport of the chemicals in the environment should also be considered. Simple proximity measures to sites of chemical releases may not adequately describe the transport of the chemical in the environment. The likely route of exposure should be considered along with the biological plausibility for an association between the exposure and disease under study. Finally, much of the environmental monitoring data was collected within the past decade and reconstructing exposure over longer periods more relevant to cancer incidence will be challenging.

Environmental databases have begun to be used in epidemiology studies of cancer to determine if disease mortality or incidence rates are higher in areas with specific environmental exposures (i.e., ecologic study designs) or as a means of classifying individuals with respect to their potential exposure in an analytic epidemiologic study design (i.e., case-control, cohort studies). With few exceptions, the residence location is used as the geographic location for assigning exposure. Below we provide an overview of the various types of spatially registered exposure data and include examples of their use in epidemiologic studies of cancer.

#### a. Water quality data

The US EPA is responsible for regulating public drinking water supplies. A water supply is regulated if it has 5 or more connections or serves at least 25 people. Routine monitoring is required for a variety of contaminants and naturally occurring elements including disinfection by-products, arsenic, nitrate, certain pesticides and volatile organic chemicals. States are required to report violations of the Maximum Contaminant Levels (MCL) to EPA. Since 1996, EPA has been required to maintain a National Contaminant Occurrence Database (NCOD) using occurrence data for both regulated and unregulated contaminants in public water systems. The majority of historical public water supply measurement data, however, reside with the states. Some states record the latitude and longitude of the locations where the water samples were taken (location in the distribution system, point of entry to the distribution system, or water source location). The location information is typically not publicly available but may be available to researchers with appropriate approvals.

The water quality data are reported by utility and to be useful for epidemiologic studies a linkage to the towns served must be established. In larger metropolitan areas multiple utilities may serve a city or, conversely, one utility may serve multiple towns and subdivisions. Therefore, establishing an accurate linkage between the study participant's addresses and water utilities is essential to avoid misclassification of exposure. Long-term exposure metrics can be calculated when a lifetime water source history is collected. Examples of studies using public supply water quality monitoring data include studies of disinfection by-products [64-66]., nitrate [67,68]., radionuclides [69,70]., and arsenic [71,72]. Contaminants such as disinfection by-products and volatile organic compounds vary in concentration across a public supply distribution system. GIS-based modeling efforts have been used to improve estimates of exposure at individual residences [73,74].

In contrast to public water supplies, private domestic wells are not regulated and there are no monitoring requirements, although well owners may be required to provide some water quality information upon the sale of a property in some states. Some states have conducted representative surveys of private well water quality [75]. A nationwide survey was conducted by EPA in 1988–1990 [76,77]. The US Centers for Disease Control (CDC) conducted a survey of coliform bacteria, nitrate, and atrazine in private wells in nine Midwestern States . The paucity of historical water quality data for private wells limits the exposure assessment for epidemiologic studies of cancer in this population.

The USGS NAWQA program has been collecting information on nutrients, pesticides, volatile organic compounds, radionuclides, and major ions in more than 50 river basins and aquifers since 1991. All of the measurement data include spatial attributes. Because the goal of this research effort is to understand ambient water quality (not necessarily the same as drinking water quality) these data may not be of direct use in epidemiologic studies. However, the NAWQA data may be useful in modeling efforts to estimate contaminant levels in private wells. EPA also maintains two data management systems containing water quality information collected by federal, state, and private groups for surface and ground waters in all 50 states. The Legacy Data Center (LDC) is an archived database with data dating from the early 20th century up to the end of 1998. STORET contains data collected beginning in 1999, along with older data documented data from the LDC. Table 4 summarizes the sources of water quality data.

**Table 4:** Sources of Water Quality data

Database name	Source Agency	URL	Geographic Resolution
National Contaminant Occurrence Database	EPA		Public water utility
National Water Quality Assessment (NAWQA) Data Warehouse	USGS		Latitude and longitude
Legacy Data Center/STORET	EPA		Latitude and longitude

#### b. Air pollutants

The EPA collects and processes monitoring data from states on six criteria air pollutants (carbon monoxide, nitrogen dioxide, ozone, sulfur dioxide, particulate matter [PM10 and PM2.5], lead) and hazardous air pollutants, of which 188 have been identified. The hazardous air pollutants (HAP), also known as air toxics, are those for which there is some evidence of an increased risk for cancer or adverse reproductive outcomes. Routine monitoring of HAPs is not required and the monitoring data that exists is sparsely distributed compared with the criteria air pollutants. The data are maintained in the Air Quality Systems database.

EPA compiles HAP emissions from stationary sources (points and areas) and mobile sources in a National Toxics Inventory (NTI) database (now combined with the National Emissions Trends data in the National Emissions Inventory database), which is updated at three-year intervals. To do the updates, EPA obtains emissions inventories from state environmental agencies and supplemental data from other sources, including the Toxic Release Inventory. The first nationwide inventory was in 1996. The spatial scale of the emissions data varies by type of source. Location information for point sources emissions is available, whereas area-source emissions are estimated at the county level. Using a dispersion model EPA has estimated the annual average HAP concentrations for each census tract in the contiguous US [78]. These datasets are summarized in Table 5.

**Table 5:** Sources of Air Quality Data

Dataset name	Source Agency	URL	Geographic Resolution
Air Quality System database	EPA		Monitoring stations (latitude, longitude)
National Emissions Inventory	EPA		Varies (point locations, county level)

Air pollutant monitoring data has been used in studies of lung cancer, which have generally employed some type of dispersion model to estimate exposure for metropolitan areas or census tracts [79-81]. Recently the modeled concentrations of HAP have been used to evaluate childhood cancer incidence [44]. Other studies have also evaluated traffic density and childhood cancer incidence [43].

#### c. Agricultural Pesticides

In the United States the U.S. Department of Agriculture (USDA) is the main federal agency responsible for collecting information on pesticide use on crops and livestock. The availability of historical agricultural pesticide use data in the US has been reviewed [82]. The first comprehensive survey of pesticide use on crops occurred in 1964 [83] and periodic surveys were conducted thereafter through the 1970s. These early surveys only provided national or regional estimates of crop-specific use for individual pesticides. From 1986 onwards, the USDA surveys produced state-specific estimates of pesticide use on field crops in the major producing states and from 1990 onwards, biannual state-specific estimates of pesticide use on fruits and vegetables were also available.

Several states have collected their own pesticide use information but most data collection efforts have been recent. Oregon enacted legislation requiring reporting of agricultural pesticide use beginning in 2002; however, insufficient funding was provided for additional years. State pesticide use data are most comprehensive for California, which has had some type of mandatory reporting for agricultural pesticides since the 1950s, currently overseen by the California Department of Pesticide Regulation. Beginning in 1969, information about restricted-use pesticides was made public. In 1990, a new law required growers to report all pesticide use on crops on a monthly basis, including the pesticide name and manufacturer, crop treated, the public land survey section where the pesticide was applied, the date and time of application, number of acres treated, method of application, and application rates. The availability of this detailed pesticide use data at the spatial scale of a section led to the development of methods to link the use data to cancer incidence data [84] for use in an ecologic study of childhood cancer at the census tract level [42]. The California data have also been used in a case-control study of pancreas cancer [85], cohort study of breast cancer [51], and an as-yet unpublished case-control study of childhood cancer. Methods have also been developed to estimate potential pesticide exposure at residences by linking pesticide use data to crop maps [86,87]. Pesticide "exposure" is assigned to homes that have crop fields within distances that reflect likely pesticide drift. Table 6 summarizes the sources of pesticide data.

**Table 6:** Sources of Pesticide Data

Dataset name	Source Agency	URL	Geographic Resolution
Agricultural Chemical Use	USDA		State
California Pesticide Use Reporting database	California Department of Pesticide Regulation		Public Land Survey Section (approximately one square mile)

#### d. Industrial releases and hazardous waste

The Emergency Planning and Community Right to Know Act of 1986 in the United States requires certain industries to report to EPA annually their releases and waste management activities involving specific toxic chemicals. The data are available to the public in a database called the Toxics Release Inventory (TRI). Manufacturing, metal mining, coal mining, and electric generating facilities must report the estimated mass of toxic chemicals released into the environment (air, water, land, or underground injection), treated on-site, or shipped off-site for further waste treatment. Reporting is required only for facilities that meet certain minimum criteria in terms of the pounds of toxic chemical produced or processed; persistent chemicals that bioaccumulate are subject to lower minimum reporting requirements. The regulations do not require environmental monitoring, so much of the data are estimates of releases. Location information is reported by the business and is not verified by EPA. Some of the strengths and limitations of these data for environmental health studies has been described [88,89].

Canada also requires reporting of emissions of chemicals rated by the International Agency for Research on Cancer as likely, probable, and possible human carcinogens for 64 industrial sectors [90]. These data form part of the Canadian Environmental Quality Database, which also contains a national inventory of municipal waste disposal sites, municipal drinking water data, air quality data, and historical industrial location and productivity data [91]. A large multi-province case-control study of 18 cancer sites was conducted with the aim of linking residential histories by postal code to the environmental database for cancer surveillance. To date, one analysis of residential proximity to 7 types of heavy industries and risk of non-Hodgkin lymphoma (NHL) has been published. Residential proximity within 3.2 km of copper smelters and <0.8 km of sulfite pulp mills was associated with an increased risk of NHL [92] after adjusting for employment in the industries evaluated. Earlier case-control studies of NHL [93] and leukemia [94] found elevated risks for residing close to industrial sites but these studies relied on a self-reported assessment of the distance of the residence from industrial facilities which may be subject to recall bias.

The EPA maintains information on the location of waste handlers, waste treatment facilities and waste sites that are regulated under the Resource and Conservation Recovery Act (RCRA) and the Comprehensive Environmental Response, Compensation, and Liability Act (CERCLA), also known as the Superfund law in the RCRAInfo database available through the Envirofacts Data Warehouse. Information on the location of companies issued permits to discharge waste into rivers is maintained in the Permit Compliance System database (also available through Envirofacts). These data sources are summarized in Table 7.

**Table 7:** Sources of Hazardous Waste Data

Dataset name	Source Agency	URL	Geographic Resolution
Toxics Release Inventory	EPA		Latitude, longitude
HazDat	ATSDR		Latitude, longitude
RCRAInfo	EPA		Latitude, longitude
Permit Compliance System (PCS)			

The U.S. Agency for Toxic Substances and Disease Registry (ATSDR) was established by Congress in 1980 under CERCLA. Since 1986, ATSDR has been required to conduct a public health assessment at each of the sites on the EPA National Priorities List, waste sites deemed to be the most hazardous. The aim of these evaluations is evaluate exposure to hazardous substances and health effects among the population living in vicinity of the site [95]. The location of the sites and information on specific contaminants by the type of media (soil, air, water) in which they were measured are available from the ATSDR HazDat database web site. Limitations of these monitoring data for cancer studies include the limited historical measurement data. A few studies have evaluated cancer incidence among those potentially exposed to hazardous waste sites [96] or municipal waste sites and incinerators [97,98].

The reconstruction of historical exposure to releases from industries and waste sites is difficult for studies of cancers of long latency. A few studies have evaluated proximity and residence duration near sites. Long duration of residence within one-half mile of a chemical plant manufacturing PCBs was positively correlated with blood serum PCB concentrations [99]. However, none of the epidemiologic studies to date determined whether proximity resulted in meaningful exposure to chemicals from the sites. Confounding by socioeconomic status should also be evaluated because manufacturing and waste facilities are more likely to be located in neighborhoods of lower socioeconomic status [100] and socioeconomic status is associated with the incidence of some cancers.

### 5. Remote sensing/aerial imaging

Remotely sensed data include images of the earth and our atmosphere obtained by satellites or aircraft. The usefulness of the information depends largely on the technology used to obtain the imagery and the additional processing that has been done to georeference the data. The USGS Earth Resources Observation Systems Data Center (EDC) is the major U.S. storehouse of these data. Aerial photography has been available since the early part of the twentieth century. Digital Orthophoto Quadrangles (DOQs) which are digital images of aerial photos which combine the image characteristics of a photo with the georeferenced qualities of a map are available through EDC from 1987 through the present. DOQs are available in black and white, natural color, or color-infrared images and have 1-meter ground resolution. Satellite imagery useful for land cover characterization includes the multispectral Landsat imagery available as early as 1972. USGS has created historical land use and land cover data derived from 1970s and 1980s aerial photography (the Land Use and Land Cover Data). A national land cover datasets (NLCD) derived from Landsat multispectral imagery for 1992 is available. The Multi-resolution Land Characteristics (MRLC) national dataset which represents land cover in 2000 is currently being developed. Table 8 summarizes these data sources. Applications of these data to studies of cancer have included mapping residences on crop maps to estimate their probable exposure to agricultural pesticides [49,87,101].

**Table 8:** Sources of Remote Sensing Data

Dataset name	Source Agency	URL	Geographic Resolution
Digital orthophoto quadrangles	USGS		1:12,000
Satellite imagery	USGS		1 meter to 1 km
National Landcover Dataset (NLCD) 1992	USGS		30 meters
Multi-resolution Land Characteristics (MRLC) 2000			

### Centralized geospatial data availability

The data sources we have described are available from a multitude of federal and state agencies. The National Cancer Institute's Geographic Information Systems web site  offers links to many of these sources, as well as links to freely available geographical tools and resources. There have also been several initiatives to try and compile spatial data into a shared, centralized information system [102]. Such centralized systems offer the promise of standardized data coding systems, file formats and geographic boundary definitions. They also facilitate the sharing of metadata, or descriptive information about the data. The leader in this endeavor has been the Federal Geographic Data Committee . The FGDC is a consortium of federal agencies with the charge of developing the National Spatial Data Infrastructure (NSDI), a set of technologies, policies, standards and procedures that facilitate the creation and sharing of geospatial data. Among the achievements of the FGDC is the establishment of the National Spatial Data Clearinghouse, a central catalog of links to geospatial data and metadata. In 2003, an enhanced web portal  was launched to further facilitate access to this data. Many states have echoed the national clearinghouse with clearinghouses of their own. The New York GIS Clearinghouse , for example, boasts over 400 member institutions providing links to thousands of datasets.

The cancer data collection community has yet to fully engage this resource. As of January 2004, no cancer incidence or mortality data was available through the national clearinghouse. The keyword "cancer" provided only a link to the Environmental Defense Scorecard, a web site from which various environmental data sets can be accessed, particularly those published by the EPA . Most of the very limited data in the "human health and disease" category accessible through the web portal consisted of hospital and other health facility locations for a smattering of states. In some cases, the steps required to make cancer data available through the national clearinghouse would be modest. For example, the NCI's mortality data, geographic boundary files, and associated metadata used in its Cancer Mortality Maps and Graphs web site are easily accessed and downloaded, and only minor modifications would be required to make them compliant with FGDC standards.

The DataWeb  is another centralized online data resource, consisting of a network of online data libraries created in a collaboration between the CDC and the US Census Bureau. The libraries consist of both microdata and aggregate data in numerous categories. Available health data includes NHANES and NHIS survey data and county-level mortality. Information in DataWeb is accessed through DataFerret, an application that prepares data sets for the user to download. It allows users to select a "databasket" of variables and then recode those variables as needed. Users develop and customize data tables and may download them to their desktop in a variety of common formats.

## Conclusion

In this article we have surveyed the distinctive characteristics of spatial data, along with commonly available sources of data relevant to etiologic cancer research. Spatial analysis is invaluable for data exploration, identification of geographic patterns, generation of new hypotheses, and providing supporting evidence about existing hypotheses. A geographic perspective will be increasingly relevant as GIS software, spatial analytic methods, and the availability and quality of geographically referenced data continues to improve.
